# Performance Factors Influencing Efficacy and Effectiveness of Iron Fortification Programs of Condiments for Improving Anemia Prevalence and Iron Status in Populations: A Systematic Review

**DOI:** 10.3390/nu12020275

**Published:** 2020-01-21

**Authors:** Anna W. Waller, Juan E. Andrade, Luis A. Mejia

**Affiliations:** 1 Department of Food Science and Human Nutrition, University of Illinois, Urbana-Champaign. 260 Bevier Hall-MC 182. 905 Goodwin Ave, Urbana, IL 61801, USA; awaller2@illinois.edu (A.W.W.); jandrade@illinois.edu (J.E.A.); 2 Division of Nutritional Sciences, University of Illinois at Urbana-Champaign, Urbana, IL 61801, USA

**Keywords:** fortification, condiments, iron-deficiency anemia

## Abstract

Iron fortification of staple foods is a common practice around the world to reduce the prevalence of iron-deficiency anemia. More recently, fortified condiments, including salts, sauces, and powders, have been tested in various efficacy trials. However, there is limited information on how nutritional, environmental, and experimental factors affect their efficacy and effectiveness. The purpose of the present work was to systematically review performance factors affecting the efficacy of condiment fortification trials. Three databases were searched using a standardized keyword search and included based on four-point inclusion criteria. Studies were evaluated against a quality assessment tool and effect sizes were calculated. Studies were ranked as low or high performing, based on whether or not they significantly improved iron-deficiency outcomes (hemoglobin, anemia prevalence, and ferritin levels). Of the 955 retrieved studies, 23 were included—of which, nine performed poorly, eight performed highly, and six were classified as neither because they did not meet the criteria of assessing the three iron outcomes. Results showed that unsuccessful trials did not consider environmental factors such as parasitic infections, nutritional factors such as micronutrient deficiencies other than iron, consumer acceptability of the product or experimental factors such as monitoring and adherence to the trials. Two common performing factors identified among those studies performing highly vs. those that did not were the control of sensory changes and monitoring of consumption compliance (i.e., dose delivery). The present work can be used as decision-making support for nutrition policy makers when determining the appropriate implementation of condiment fortification programs.

## 1. Introduction

Iron deficiency and iron-deficiency anemia are the most prevalent and widespread nutritional conditions around the globe at all socioeconomic levels. The deficiency of iron affects children and women of reproductive age primarily, especially during pregnancy [1]. This condition is linked to poor neurological and cognitive functions in children, to impaired work capacity in adults, and greater mortality in pregnant women [2]. According to the World Health Organization (WHO), the latest prevalence estimates of anemia in 2016 were 41.7% in children and 32.8% in women of reproductive age, being most prevalent in low- and middle-income countries [3]. Practically no progress has been made in the last seven years since 2012 when the United Nations World Health Assembly set a goal for lowering the prevalence of anemia by 50% in women of reproductive age by 2025 [4]. According to the WHO global health observatory, the prevalence of anemia in women was 30.3% in 2012 and 32.8% in 2016, while the prevalence of anemia in children (<5 years) has remained unchanged at 41.7% during the same period. [3]. This high anemia prevalence indicates a serious public health concern, particularly for children. Iron deficiency has been attributed to insufficient iron intake, consumption of plant-based diets containing inhibitors of iron absorption, and infections, especially malaria and hookworms [5].

Several strategies have been used throughout the years to combat iron deficiency, including the promotion of dietary diversification with iron-rich foods, iron supplementation of women of reproductive age, and fortification of staple foods with iron [5]. Since 1994, the World Bank has recognized food fortification of staple foods as the most cost-effective intervention for improving lives and human development in a relatively short time [6]. Furthermore, in 2008 the Copenhagen Consensus identified food fortification as one of the most cost-effective public health interventions for best health and nutrition outcomes [7]. Following this strategic direction, continuing efforts have been made to eradicate iron deficiency by fortifying, with this essential mineral, staples such as cereal flours and more recently, regional foods consumed in certain parts of the world, such as condiments and seasonings, whose consumption is expanding globally [8].

The fortification of seasonings and condiments have a particular interest as these are consumed locally and are culturally adapted to the population. Therefore, populations are expected to demonstrate greater adherence to the interventions [9]. Current food vehicles for iron fortification include soy sauce, fish sauce, table salt, flavoring powders, curry powder, and bouillon cubes, among others [8]. This modality of fortification has been evaluated in Asian and African countries. In some cases, technologies have been fully evaluated for their efficacy and effectiveness, and in limited cases, these are commercially available, or manufactured by the industry for government programs, in countries like India, China, Cambodia, Bangladesh, Thailand, Vietnam, and Indonesia [9], and more recently, in Argentina [10].

When assessing the efficacy and effectiveness of food fortification interventions, the outcome is often influenced by several nutritional, environmental, and experimental factors. They include health status, type of infection, parasitism, rate of anemia at baseline, quality of the diet, duration of the study, monitoring of compliance, adherence to the programs, reigning diet, education and communication components, and even deficiencies of other micronutrients such as vitamin B12 and/or folate, among others [11]. Nonetheless, the relative impact of these factors has not been comprehensively evaluated. The efficacy of iron fortification of already assessed condiments on increasing hemoglobin levels and decreasing the risk of anemia is well-established, particularly for double-fortified salt with iodine and iron [12,13,14]. The effectiveness of these interventions is less documented and, although hemoglobin and ferritin levels may slightly increase, in some instances the prevalence of anemia does not change in the population [13,14]. Furthermore, results regarding the role of potentially influencing factors affecting the outcome of the interventions are often mixed, show no effect or are not reported [12,13]. This limited information justifies a more comprehensive analysis of the interplay and degree of impact of associated conditions influencing the performance of interventions with iron-fortified condiments, hoping it will contribute toward their optimization. Therefore, the objective of this systematic review is to conduct a comprehensive assessment of performing factors impinging, negatively or positively, on the outcomes of iron fortification of condiments and seasonings in studied populations, particularly on their impact on anemia prevalence and hemoglobin and ferritin levels. The populations investigated consisted of children, women and general population, with different degrees of anemia, iron deficiency or both. Within this context, we also wanted to know whether established iron-deficient individuals could benefit from iron fortification of condiments by lowering anemia prevalence and increasing iron stores, once performing factors are identified and optimized.

## 2. Materials and Methods

### 2.1. Search Strategy

Experts (*n* = 2) on novel fortification strategies were initially contacted to collect potentially relevant literature. The Pubmed, Web of Science, and Scopus databases were searched by using the following keyword search: ((fortif *) AND “iron” AND (Condiments [MesH] OR Seasoned OR Seasoning OR Bouillon * OR “Soy sauce *” OR “Fish sauce *” OR Powder * OR “fortified salt *”)) NOT (“milk powder *” OR Noodle * OR “home fortification”)). A language filter was applied to specify only English and Spanish studies. Other languages were not considered. Once articles were collected, literature was back-searched to identify additional references that met the inclusion criteria. Review articles were used for back-searching references.

### 2.2. Study Selection Criteria

Articles were assessed by title and abstract to include or exclude those based on the following criteria: Include articles that assessed efficacy, effectiveness, anemia prevalence, hemoglobin, or serum ferritin outcomes; studies assessing iron-fortified condiments (sauces, curry powders, bouillon cubes, salts, spices, seasonings, MSG); human studies; randomized or quasi-randomized controlled trials (either individual or cluster-level), cross-over trials, non-randomized controlled trials, clinical trials, community-based studies and prospective observational studies with a control group. Exclude articles not pertaining to the aforementioned fortification strategies; articles published prior to 1999; behavior change only outcome studies; animal studies; survey and consumption data, in vitro studies, or case studies. Articles were collected with publication dates in the last 20 years, January 1999–May 2019, to collect the most relevant and new information. All populations were considered, including children, women, iron-deficient populations, anemic populations, and general populations.

### 2.3. Data Extraction

Data were extracted from included articles into a Microsoft Excel table on the following characteristics: publication year/author/journal, study type, study aim, condiment type, iron source (and preparation of condiment), sample size, duration of trial, population (age, gender, physical status (pregnancy, severity of iron deficiency, infection), diet, geography (country, rural/urban) including setting of trial (school, home, refugee camp, etc.)), performance factors (education framework (if identified; i.e., Bronfenbrenner’s ecological framework, the community-based participatory research (CBPR) framework, the social cognitive theory, theory of planned behavior, the health belief model, peer learning theory), communication with participants, behavior change component, policies/legislation, parasite treatment/no treatment, access/coverage, delivery mechanisms, monitoring scheme, adherence to treatment/insufficient or sufficient consumption of fortified condiment, sensory, monitoring compliance, waste, consumption drift, outcomes (anemia, serum ferritin, hemoglobin, hematocrit, mean corpuscular volume, total iron binding capacity, C-reactive protein, serum iron, serum soluble transferrin receptor, morbidity outcome: diarrhea, number of days sick, mortality, any reported adverse effects). In the case of reviews or other article types not providing the relevant characteristics to extract, N/A was used as a placeholder.

### 2.4. Study Quality Assessment

The included studies were assessed for quality and risk of bias using the guidelines found in the ‘Quality Criteria Checklist: Primary Research’ of the Academy of Nutrition and Dietetics Evidence Analysis Manual [15]. This comprehensive tool was designed for food and nutrition interventions [16]. The Quality Criteria Checklist for primary research consists of 10 questions. Depending on the type of design, each question has several sub-questions (*n* = 2–8) to guide the rating. One author (JEA) assessed the quality of the articles at the study level on clarity of the research question, aspects of selection (random sequence generation and concealment of allocation methods) and comparability of study groups, retention rate (completeness of outcome data), detection (blinding of outcome assessors), performance (blinding of participants) and validity of outcome measures and any intervening factors, quality of statistical analysis and conclusions made with consideration of study limitations and likelihood of sponsorship bias. Any emerging conflicting assessments were discussed with an independent reviewer. Manuscripts were rated as positive (+), neutral (0), or negative (−). The quality assessment was not used to exclude studies from this review, but to support the discussion of performing indicators.

### 2.5. Data Synthesis

Given that the methods used for assessing change in hemoglobin varied across studies (i.e., control or baseline to endpoint), change in hemoglobin between baseline and endpoint were expressed as a standardized mean difference (effect size) to compare all studies based on the same measure. The effect size on hemoglobin levels was calculated using an online software tool based on the difference between the two means at the end of the intervention period, divided by the pooled estimate of standard deviation [17]. This helps calibrate the difference between the experimental and control groups in terms of the standard deviation. Effect sizes are used to compare studies to one another, but not to categorize studies based on effect sizes [18]. This is commonly conducted in meta-analysis reviews and reported as an odds ratio or relative risk [19,20]. The effect sizes can be deemed small (≤0.2), medium (≥0.2–<0.8), or large (≥0.8), based on previous cutoffs [20].

## 3. Results

### 3.1. Study Selection

**Figure 1:**
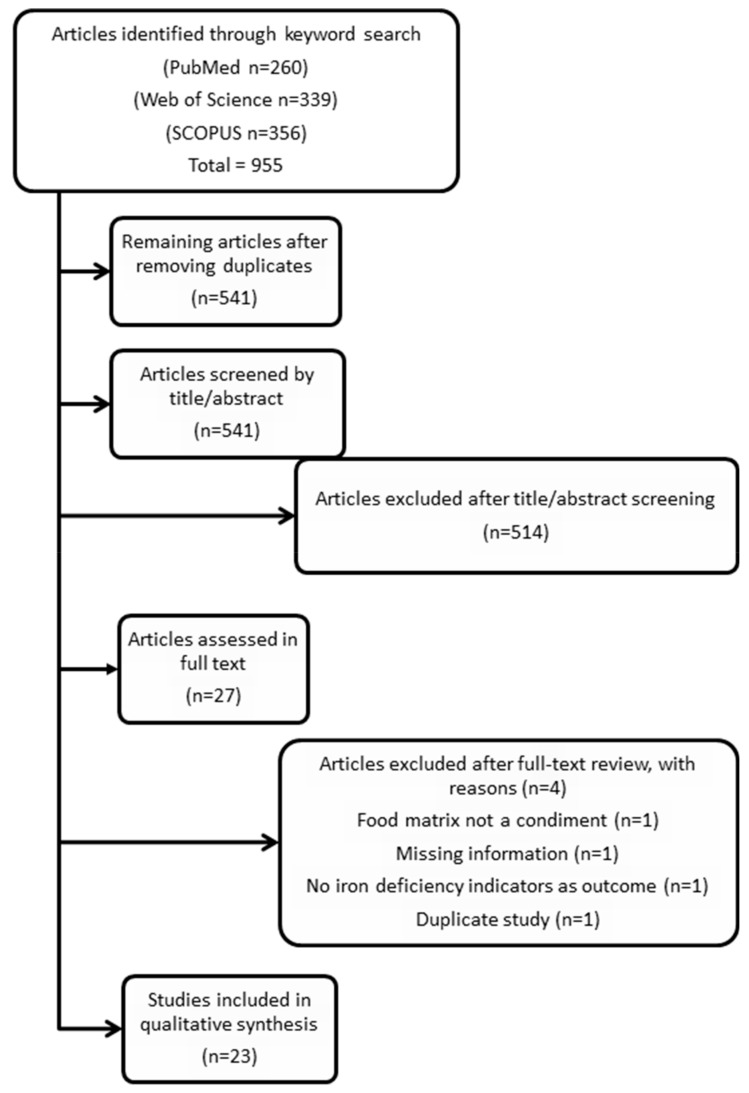
PRISMA study selection flow chart.

In the initial search of the literature (first phase), 955 studies were identified from Pubmed (*n* = 260), Web of Science (*n* = 339), and Scopus (*n* = 356) databases. After duplicates were removed, 541 studies remained. In the second phase, distillation was conducted by two authors (AWW and LAM) who reviewed the 541 studies by title and abstract and agreed to exclude 514 papers. After the distillation phase, 27 papers were reviewed full-text in the final third phase. After independent full-text review, the reviewers (AWW and LAM) met to discuss the exclusion of four articles under doubtful deliberation. A final agreement was reached based on the following reasons: food matrix not a condiment (*n* = 1), missing information (*n* = 1), no iron deficiency indicators as outcomes (*n* = 1), and duplicate study (*n* = 1). A total of 23 papers remained for the data extraction, study quality assessment, and data synthesis stages (see Figure 1).

### 3.2. Data Analysis

The selected twenty-three studies [21,22,23,24,25,26,27,28,29,30,31,32,33,34,35,36,37,38,39,40,41,42,43], two including subgroups of children and adults [22,42], were then evaluated for the impact of the iron fortification interventions on changes in hemoglobin, the prevalence of anemia and serum ferritin levels. An effort was made to identify negative and positive performing factors that may have influenced the outcomes. As presented in Table 1, the most tested condiment, as a vehicle for efficacy or effectiveness of iron fortification, was salt. This fortification approach included 13 Double-fortified Salt (DFS) [21,22,23,24,25,26,27,28,29,30,31,32,33] and five Multiple Micronutrient Fortified Salt (MMFS) studies [34,35,36,37,38]. Only one study evaluated a seasoning powder [39], two studies evaluated fish sauce [40,41], and two studies evaluated soy sauce [42,43]. The majority of studies evaluated the efficacy of the interventions, and only two [41,42] assessed effectiveness. There were no efficacy or effectiveness studies of iron-fortified bouillon cubes. All studies were conducted in Asia or Africa using as subjects children or women of reproductive age during different time lengths using as fortificants a variety of iron forms that included micronized ground ferric pyrophosphate [21,30,33,38], encapsulated ferrous fumarate [21,31], ferrous fumarate [22], ferrous sulfate [23,28], microencapsulated ferrous fumarate [24], unknown iron source [25,26,27], ferrous sulfate monohydrate chelated with malic acid and sodium hexametaphosphate [29], ferrous sulfate hydrate encapsulated with partially hydrogenated vegetable oil [32], chelated ferrous sulfate [34,35,36,37], H-reduced elemental iron encapsulated with partially hydrogenated vegetable oil [39], ferrous sulfate citrate [40], or NaFeEDTA [40,41,42,43]. Blood hemoglobin was the main hematological indicator in all studies to evaluate the effect of iron fortification. Some studies reported anemia prevalence and only a few analyzed serum ferritin (with or without correction for inflammation). The majority of DFS and MMFS studies assessed urinary iodine excretion (UIE) and verified that iron fortification did not jeopardize iodine fortification. Furthermore, in order to assure the success of the interventions, some studies included as part of the research protocol, the monitoring of potential performing factors such as an education component, assessment of behavioral changes, parasitic treatment, assessment and correction of micronutrient deficiencies, dietary intake, sensory and acceptability evaluation of the product and adherence to the programs. Sensory evaluation and product acceptability were of foremost importance, since some studies reported changes in the color of the DFS and of some foods when DFS was used for cooking [22].

The level of success of iron fortification for increasing hemoglobin, correcting anemia, or improving iron status was not the same for the different studies. Table 2 presents the distribution of responses in changes of hemoglobin, anemia prevalence and ferritin levels in children, women of reproductive age and the whole population at the end of the interventions. Out of 16 children studies, 13 showed increases in the levels of hemoglobin, and there was no change in three of them. In women, six out of eight studies resulted in an increase in hemoglobin and there was no change in one. Of the one study covering the whole population, only one experienced a significant increase in hemoglobin. Anemia in children was reported only by 12 studies, 10 of them decreased the prevalence of this condition and two reported no change. Out of seven studies in women that reported data on anemia, its prevalence decreased only in four studies and there was no change in three. In the one study covering the whole population, anemia was not assessed. Regarding serum ferritin as an indicator of iron deficiency, only 11 out of the 16 studies in children reported data on this parameter showing an increase in nine studies and not change in two of them. In women, four studies showed an increase in ferritin and there was no data in the remaining four. In the whole population, the study did not assess ferritin levels. A general observation in all these studies was that often hemoglobin and/or ferritin levels increased but the prevalence of anemia did not decrease.

We then investigated, based on information provided by the authors, why nine of the studies in Table 2 did not experience changes in the right direction—that is, increasing hemoglobin, lowering the prevalence of anemia or increasing serum ferritin, in either children, women or in the whole population. Such studies, associated with potential negative performing factors that may have jeopardized reaching optimal outcomes, are presented in Table 3. Based on the comments made by the authors, there were organoleptic changes in two of the studies, consisting of a change in color of the iron-fortified salt or changes in the color of food when cooking using DFS as an ingredient [22,30]. It was believed that these color changes of the food vehicle decreased the acceptance, and thus the consumption of the fortified product impacting the outcome of the interventions. Two other studies were believed to be impacted negatively in the iron outcome due to an insufficient amount of iron in the fortified product to fulfill iron requirements, especially in women [25,39]. Two studies reported environmental conditions, such as the presence of malaria and parasitism, as factors influencing the outcomes negatively [27,30]. Two other reported deficiencies of micronutrients such as folate and vitamin B12 as possible causes for not attaining a significant reduction in anemia prevalence [24,30]. On the other hand, three of these studies did not reach statistical significance in one or more iron parameters possibly due to statistical issues such as an uneven balance in the prevalence of anemia between experimental and control group at baseline or increases on iron parameters in control groups at the end of the interventions [22,35,37].

There was no apparent association between the type of iron form used and the outcome in iron parameters, except when using FeNa-EDTA in soy and fish sauces, which showed a greater positive response of the intervention.

### 3.3. Study Quality Assessment

All twenty-three studies were assessed using a 10-point quality assessment tool [15] and classified based on their responses to being strong, moderate, or weak studies. The full quality assessment table can be seen in Supplementary Materials Table S1. There were *n* = 12 studies classified as strong [21,24,30,31,32,33,35,38,39,40,41,42], *n* = 7 studies classified as moderate [22,26,28,29,36,37,43], and *n* = 4 studies classified as weak [23,25,27,34].

### 3.4. Data Synthesis

**Figure 2:**
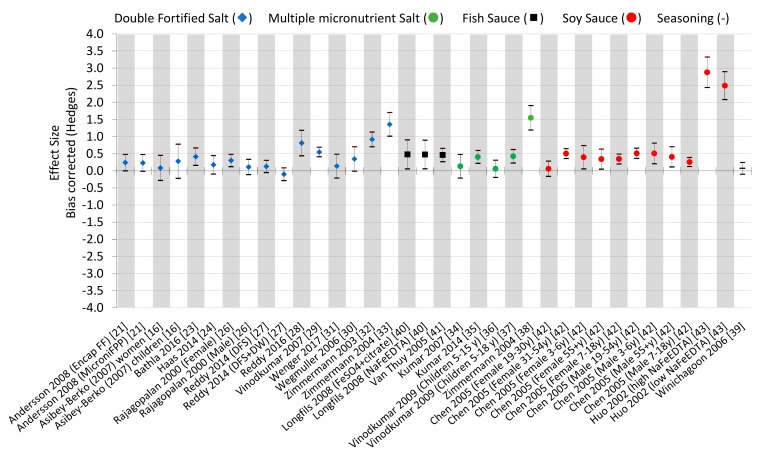
Effect sizes of studies included in this review. The effect size was bias-corrected using the Hedges estimation. When a similar study is included, more information in parentheses indicates the difference. Double-fortified salt (DFS), fish sauce, soy sauce, multiple micronutrient salt (MMS), fortification. Deworming (DW), ferrous fumarate (FF), and ferric pyrophosphate (FPP).

Effects sizes were estimated for each study, disaggregated by gender, age, iron source, and/or iron concentration when possible (Figure 2). Effect sizes were considered negligible if the standard deviation crossed the 0 x-axis, which was seen in nine studies [22,24,26,27,31,34,36,39,42].

Interestingly, Rajagopalan et al. [26] demonstrated a negligible effect size for men, but a positive effect size for women, indicating that double-fortified salt may be a more effective treatment for women than men. DFS fortified with micronized ferric pyrophosphate showed the highest effect size for this condiment at 1.4 [33], indicating that this iron source may contribute to its greater effect sizes over the other iron sources used for these studies. Seven of the 16 DFS data points (44%) showed negligible effect sizes. Organoleptic changes of the DFS could be associated with weak effect sizes.

Multiple micronutrient fortified salts showed mixed effect sizes. Most notably is the 1.55 effect size using micronized ferric pyrophosphate, vitamin A, and iodine in triple fortified salt [38]. Kumar et al. (2014) also showed a positive effect size (0.4) using chelated ferrous sulfate [35]. However, Kumar et al. (2007) showed a negligible effect size (0.13) using chelated ferrous sulfate amongst vitamin A, B1, B2, B6, B12, folic acid, niacin, calcium pantothenate and iodine [34], as did Vinodkumar 2009 for children ages 5–15 years using the same combination and sources of micronutrients [36].

Both studies that assessed fish sauce showed positive effect sizes. Interestingly, similar effect sizes were seen for fortified fish sauce using either NaFeEDTA or FeSO_4_ + citrate, indicating that either iron source can be used for positive effects in fish sauce [40,41].

The fortification of soy sauce showed the highest effect sizes [42,43]; all of the age/gender groups showed considerable effect sizes, with the exception of women 19–30 years of age [42]. Nonetheless, even at a low concentration of NaFeEDTA, the large effect size of 2.5 indicates the strength of this condiment as a fortification vehicle [43].

Seasoning powder demonstrated a negligible effect size (*n* = 1). However, more studies will need to assess this vehicle as a fortification vehicle before complete conclusions can be drawn [39].

Regarding the studies assessing iron-deficient populations (*n* = 4) [23,30,40,43], the average effect size was 1.18. On the contrary, studies assessing a general population (not necessarily iron deficient, *n* = 18) [21,22,24,26,27,28,29,31,32,33,34,35,36,37,38,39,41,42] had an average effect size of 0.39. This difference illustrates that iron-deficient populations show a relatively larger effect size because iron is absorbed more readily than in the general population.

Adherence and/or compliance was addressed by 15 studies, having an average effect size of 0.52 and those that did not address adherence or compliance (*n* = 7) had an average effect size of 0.67. This relative difference is not large, indicating a negligible effect between study outcomes that did or did not address compliance and adherence.

## 4. Discussion

Our review of studies conducted in the last 20 years revealed that during this period, there were 21 studies that assessed the efficacy of iron fortification of condiments in children and adults and only two studies evaluated the effectiveness of this type of intervention on the whole population [41,42]. Most of these studies used salt fortified with iodine and iron or salt fortified with multiple micronutrients. Only a limited number of studies were conducted using other vehicles such as soy and fish sauces. Various forms of iron had been used with the objectives of increasing bioavailability and maintaining the quality and stability of the fortified product, the iron compound and the levels of iodine in salt. Iron forms used included chelated ferrous sulfate, ferrous fumarate, iron pyrophosphate, and in some instances in microencapsulated forms to prevent unwanted interactions between iron and iodine that could jeopardize product quality and organoleptic properties of the fortified product. There has been no evaluation of bouillon cubes and there was only one assessment of a fortified powder when used as a condiment in food preparation. All studies were conducted in Asia or Africa, where the potential of fortifying condiments seems more promising due to culture and tradition. The main iron indicators monitored were the levels of hemoglobin, the prevalence of anemia and in some studies, the levels of serum ferritin.

As shown in Table 2, there was a variety of responses in iron indicators as a result of the iron fortification intervention in children, women, and the whole population. In some cases, the level of hemoglobin did not increase, and in others, the level of hemoglobin increased significantly but the prevalence of anemia did not decrease. Likewise, serum ferritin, as an indicator of iron deficiency, did not always increase. Interventions with children showed the most consistent positive efficacy result. In these studies, 13 out of 16 studies increased the levels of hemoglobin and nine out of 11 studies that measured ferritin showed a significant increase in this indicator. This observation was particularly more evident in children studied and maintained under confined conditions like schools or childcare centers, where monitoring and compliance are easier to control. While it seems that adherence had no effect on the study effect sizes, whether or not the population was iron-deficient at baseline showed an increase in the effect size. Thus, it is hypothesized that a large-scale effectiveness trial using iron-fortified condiments without monitoring adherence and compliance may not see a difference in results than its monitored counterpart. However, further studies are needed to prove this point. Additionally, it should be noted that iron-fortified condiments targeting a specific iron-deficient subgroup may see more preferable results than the general population. Policy makers should understand the risks of non-targeted individuals being exposed to iron fortification during targeted trials, specifically those individuals that might possess the thalassemia trait in which iron overload is a risk.

We identified nine studies in which one or more iron parameters did not change in the positive direction as a result of the iron interventions, either not increasing hemoglobin significantly, not lowering the prevalence of anemia, or not increasing serum ferritin (Table 3). Upon analysis, several of these studies using salt as a vehicle reported lower acceptability of the fortified product due to organoleptic changes. Those reporting changes, made particularly by women, included a different color appearance of the fortified salt or changes in the color of the food when cooking using fortified salt as an ingredient. Organoleptic changes of DFS, particularly color changes of the fortified product, have been reported by several investigators [22,32], and efforts are being made to correct or at least alleviate this unwanted phenomenon [11]. Visual and flavor modification instead influences the acceptability of the product with a consequential decrease in consumption of the fortified product lowering the iron intake. In addition, two of these nine low-performing studies reported an insufficient amount of iron in the fortified product to meet iron requirements as a cause of not improving iron deficiency. This observation coincides with the potential decrease in the consumption of fortified salt due to organoleptic changes that would also lead to lower iron intake. The need for adding meaningful amounts of fortificants to staple foods and their adequate consumption to fulfill dietary gaps has been demonstrated in successful fortification programs in Latin America, including fortifying foods with iron [44]. In some of these studies in Table 3, negative performing factors reported by the authors were also deficiencies of other micronutrients important in anemia, such as folate and vitamin B12. The lack of folate and vitamin B12 impairs the production of red blood cells and therefore the persistence of anemia despite adequate iron nutrition [45]. Furthermore, as suggested by two of these studies [27,30], environmental factors such as the presence of high parasite burden, particularly from hookworms, as well as malaria, can also contribute to the development of anemia by producing intestinal iron losses and red cell hemolysis, respectively [46]. One systematic review also found that iron supplementation, in addition to deworming of schoolchildren, showed greater changes in hemoglobin and reduction of anemia than studies that just dewormed (*n* = 8 studies) [47]. An interesting case was seen in one of the included studies, Reddy et al. (2014) [27] where anemia prevalence significantly decreased (−6.3%) among participants consuming DFS and who were dewormed at baseline, but did not significantly decrease (+1.5%) among those who only consumed DFS and were not dewormed at baseline. Nonetheless, this finding is contradictory to a systematic review and meta-analysis conducted by Ramírez-Luzuriaga et al., in which they found deworming at baseline not to be a significant predictor of the risk of anemia and hemoglobin concentration response to DFS interventions [14], and thus requires further investigation.

We then wanted to examine the other end of the spectrum, the most successful iron fortification interventions, as detailed in Table 4. That is, those studies that showed changes in all three iron indicators, (increased hemoglobin, decreased anemia prevalence and increased serum ferritin) in the right direction indicating clear efficacy of the iron fortification. We wanted to identify what was done in these particular studies associated with their success, particularly different or contrasting activities to the ones conducted in the less successful studies shown in Table 3. It was revealed that out of eight studies in this category, we found that five were carefully monitored in all aspects of the intervention, particularly the sensory characteristics and acceptability of the fortified product. This was especially important considering the changes in color in iron-fortified salt and when salt is used for cooking. The studies included in the present work using fortified soy and fish sauce did not report any taste acceptability issues. This observation supports the argument that changes in color and/or flavor of the fortified product are critical determinants of acceptability. Due to their distinctive dark brown color and strong flavor, the sauces used in these investigations masked any potential changes in color that could be produced by the addition of iron. In support of this notion, a study conducted in Thailand found good similar sensory acceptability when fortifying soy sauce with NaFeEDTA [48]. Although a metallic taste has been associated in some instances with the fortification of soy sauce with NaFeEDTA, in a systematic review by Huo et al. (2002), only one of 16 studies conducted in China assessing NaFeEDTA-fortified soy sauce reported negative taste effects [49]. In addition, the authors showed a pooled weighted mean difference in hemoglobin of 0.88 g/dL and a decrease in anemia prevalence of 25%, indicating a clear efficacy of these types of interventions. These findings illustrate the significant potential of reducing iron-deficiency anemia prevalence within an appropriate implementation context for condiments.

Five of these studies also monitored and corrected as needed, micronutrient deficiencies, in particular vitamin A. The positive role of vitamin A in iron utilization has been documented since 1998 [50]. Only three of these studies conducted parasitic treatments of the subjects previous to the intervention. While all studies presented in Table 4 demonstrate positive effects in the three iron indicators extracted (hemoglobin, anemia prevalence, and serum ferritin), it should be noted that hemoglobin and serum ferritin are the best indicators of iron deficiency. Anemia prevalence can be an indicator of iron deficiency or other external factors such as other micronutrient deficiencies or parasitic infections. Thus, when analyzing randomized controlled trials, an outcome of lowered anemia prevalence can be attributed to a holistic treatment within the study design.

It needs to be indicated that the remaining six studies that did not conform to the low-or high-performing criteria [23,26,29,34,40,43] showed increases in hemoglobin but did not assess or report anemia, ferritin or both. Therefore, such studies were not classified as high performers based only on the criteria of lacking positive changes in the three parameters.

The purpose of the quality evaluation was to assess the internal validity of the studies, as well as to appraise the quality of the study design. This quality assessment was not used to exclude studies, but to add a layer of internal validity to the already extracted data. The degree of internal validity of study design is used in order to warn the validity of successful trials when it is presented that internal validity is also threatened. For example, Huo et al. showed the highest effect size of all 22 studies included for the effect size calculation (2.88 and 2.49, high and low NaFeEDTA, respectively) [43]. This iron fortificant is known for its higher bioavailability and limited effect on the organoleptic properties of food [51]. Despite these findings, this study [43] was rated as moderate in the quality assessment due to a lack of blinding to prevent bias and inadequate description of statistical analyses. Therefore, its results must be cautiously analyzed, given these reasons for doubting its internal validity. Similarly, Reddy et al. (2016) showed a large positive effect size (0.81) using ferrous sulfate fortified DFS with pregnant women. However, blinding and statistical analyses (e.g., power calculations, ANOVA assumptions) were not used or described in the study design [28]. Blinding is a critical component in randomized, placebo-controlled trials [52]. In the case of those studies using salt that is visibly different from the control, any attempt to blinding will be difficult to accomplish. Thus, it is important that the investigator acknowledges this as a limitation. Many of the studies included in this review did not use blinding, and some that reported using it did not indicate how blinding was operationalized. These examples warrant caution when extrapolating results that might support policy decisions.

On the other hand, studies that showed positive effect sizes also had strong internal validity from the study quality assessment. These studies include [32] (DFS, 0.91), [34] (DFS, 1.36), [38] (MMS, 1.55), [40] (fish sauce, 0.46), [41] (fish sauce, 0.48), and [42] (soy sauce, 0.41 on average) [32,33,38,40,41,42]. Interestingly, all of the high-performing studies included in Table 4 also were classified as strong from the quality assessment. Thus, when rationalizing a fortification program using condiments as delivery vehicles, these studies illustrate the benefits of designing a strong efficacy study along with adequate monitoring and control of influencing covariates.

It is also important to mention the content of sodium that participants were consuming in the condiment fortification trials. In the salt trials, participants consumed an average of 10 g of salt per day. In the soy sauce and fish sauce trials, participants were also consuming 10 mL of sauce per day. Sodium content in soy sauce ranges between 1.9 and 5 g/100 g [53]. Sodium content in the fish sauce varies, but one study reported 3672 ± 580 mg/100 g [54]. The US Adequate Intake for sodium is 1.5 g/day for healthy individuals aged 9–50 years and 1–1.2 g/day for children 1 to 8 years [55]. The level of salt consumption as found by these evaluated fortification studies may lead to excessive amounts of sodium intake, not aligning with the WHO’s non-communicable disease-related target (<5 g salt per day) of reducing salt/sodium intake from current consumption levels by 30% [56]. Nonetheless, no adverse events, related or not to the level of salt intake, were reported by the only one of all reviewed studies who monitored adverse events in the population under investigation [42]. Therefore, the authors recommend the collection and reporting of adverse events within future fortified condiment trials.

Sodium intake continues to be high throughout most of the world, leading to increasingly high rates of hypertension [57]. As such, in populations where the prevalence of hypertension is dangerously high, condiments that are excessive in sodium should be cautioned when used as vehicles in fortification programs. Education and proper communication of benefits and associated risks are critical in program design and implementation.

This review has several limitations that require disclosure. A large proportion of the included studies were on the ability of DFS to address low hemoglobin or anemia, especially in India. A smaller proportion of included studies consisted of other vehicles such as sauces and seasoning powders. Most of the studies included were from India (*n* = 13) and African countries (Ghana, Morocco, and Cote d’Ivoire), but none from the Americas, Oceania, or Europe; thus, compromising the generalizability of the results to the whole Sub-Saharan Africa and other continents. Most of the studies included in this review were RCTs. Nonetheless, most studies lacked information on program implementation such as randomization, blinding, monitoring, and formative evaluation, which adds to the problem of abstracting and describing poor or good performing factors. Finally, this review included studies only published in English. This is a known limitation for most systematic reviews. However, we considered the findings of articles in other languages as these were included in other systematic reviews, such as in the case of using NaFeEDTA to fortify soy sauce as reported by Huo et al. [49], which included 16 articles, the majority in Chinese.

## 5. Conclusions

The present review systematically collected and assessed the efficacy of 23 condiment-based iron-fortification trials conducted in the last 20 years and their associated performance factors. The internal validity of the study designs was assessed via a quality assessment, and effect sizes were calculated. Overall, it was found that effective condiment vehicles for fortification are those that can mask the organoleptic changes due to the physical characteristics of the fortified food, such as fish or soy sauces. These condiments also mask changes in taste due to their inherent strong flavor. The efficacy of fortified salt is well proven and can help to address iron deficiency. However, it is best when using an iron source that does not interfere with other fortificants (e.g., promote oxidation) and does not produce adverse organoleptic changes, as is the case of micronized ferric pyrophosphate. For best results, programs should rigorously address monitoring compliance and adherence. Furthermore, deworming is necessary to enhance the efficacy of programs implemented in areas with a high parasite burden. Studies are needed to determine the efficacy and effectiveness of bouillon cubes (*n* = 0 studies), curry powders (*n* = 0 studies), and seasoning powders (*n* = 1 study) before conclusions can be drawn for these vehicles. Special attention should be given to those studies that show positive effect sizes as well as a strong internal validity of study design, and caution to those studies that show positive effect sizes but with moderate or weak study design. This review can be used to advise policy makers and those in decision-making positions on best practices and protocols for condiment fortification programs. If implemented correctly, fortifying condiments is a potential nutrition-specific strategy for governments and NGOs to address iron deficiency in at-risk populations.

## Figures and Tables

**Table 1:** Data extraction for systematically selected 23 studies.

Study/Objective/Country/Duration and Experimental Design/Subjects	Condiment/ Fe Source/Concentration	Iron Intervention Outcomes (Hemoglobin, Anemia Prevalence, and Ferritin)	Selected Performance Indicators				
			Education/Behavior Change Component	Parasitic Treatment	Sensory/Acceptability Testing	Monitoring Compliance/Adherence	Other Micronutrients Associated with Anemia
Andersson et al., 2008 [21] Compare the efficacy of DFS with FePP and EFF India 10 mo RCT 458 rural children (5–15 y)	DFS Micronized ground FePP or encapsulated FF 2 mg Fe/g salt	Hb (+0.6 * and +0.8 * g/dL, FePP and FF) Anemia prevalence (−9.1% * and −10.1% *, FePP and FF) Ferritin (+6.4 * and +4.9 * μg/L, FePP and FF)	At each salt distribution, families were instructed that the new salt should be used for all cooking and food preparation. Health effects were explained to noncompliant families.	Albendazole treatment at 1 mo and 8 mo	Sensory panel (*n* = 18 women). Adverse color changes in cooked foods using FF. Acceptability testing pre- and post-study with women at a local market (*n* = 50 women)	Salt container was weighed on four consecutive mornings (*n* = 80 households pre-study; *n* = 85 households post-study). Salt intake per person was 11.3 ± 5.1 g/d throughout the 10 mo study period. 17% of households (*n* = 20) in the FF group stopped using the salt for short periods at some point during the trial. Salt consumption was resumed after beneficial health effects were explained.	Vitamin A supplements (200,000 IU) distributed at 1 mo
Asibey-Berko et al., 2007 [22] Test the efficacy of DFS on anemia and iodine deficiency of women and children Ghana 8 mo RCT 300 NP, NL women (15–45 y) and 157 children (1–5 y)	DFS FF 1 mg Fe/g salt	No Hb post-treatment data Anemia prevalence (−21.7% * and +3.3%, children and women) Ferritin NA	NA	NA	2.7% of the women in the DFS group reported darkening of fried plantains with DFS	Weekly household visits to monitor salt usage, encourage compliance, and check health status (diarrhea, pregnancy, malaria, constipation). No adherence data were available.	NA
Bathla et al., 2016 [23] Evaluate DFS effect on nutritional status of anemic children India 90 day pre- and post-test design 120 children with Hb <12 g/dL (7–9 y)	DFS FS 0.85 mg Fe/g salt	Hb (+0.6 * g/dL) Anemia prevalence (−6.6% *) Ferritin NA	NA	NA	NA	Supervision of a researcher during mid-day meal. Adherence NA.	NA
Haas et al., 2014 [24] Test the efficacy of DFS in reducing ID in WRA India 7.5–9 mo RCT 212 NP women (18–55 y)	DFS Micro-encapsulated FF 1.1 mg Fe/g salt	Hb (+0.24 * g/dL) Anemia prevalence (+1%) Ferritin (+0.13 * log10 μg/mL)	NA	200 mg of albendazole at −4 wk and at study midpoint	Stability, organoleptic, and acceptability testing of the DFS showed some discoloration and black specks, but the salt was still considered acceptable for consumption by all of the participants	Salt consumption was monitored at midpoint and endline by weighing salt bags, salt consumed at major meals, and dietary recalls. The salt consumption per person was 12.4 ± 7.9 g in the DFS group and 15.0 ± 13.6 g in the control group.	Folate deficiency (−13% *) Vitamin B12 deficiency (+9% *)
Nair et al., 2014 [25] Test efficacy of DFS with nutrition health education on pregnant, anemic mothers India 2 mo RCT 135 pregnant, anemic women	DFS Fe source NA 1 mg Fe/g salt	Hb (−0.15 g/dL) Anemia prevalence NA Ferritin NA	Nutrition Health Education (NHE) specifically pertaining to the use and storage of DFS, iron-rich foods, consequences of deficiency.	NA	NA	Compliance monitoring NA NHE improved adherence to salt consumption by 42%.	NA
Rajagopalan et al., 2000 [26] Determine if DFS improved the Hb and productivity of tea pickers India 1 y RCT 793 tea pickers	DFS Fe source NA 1 mg Fe/g salt	Hb (+0.85 * and 1.55 * g/dL, men and women) Anemia prevalence (−22.5% and −15.5%, dewormed and not dewormed) Ferritin NA	Education on DFS nutritional benefits, cooking demonstrations. DFS was introduced for 3 mo prior to the study to allow for behavior change.	Dewormed half the population at baseline	Through cooking demonstrations, they observed that the fortified salt did not change the color, taste, or appearance of the food	Periodic surprise visits to the houses of the workers and collected salt samples from their kitchens for analysis. These checks proved that the population was using only Nutrisalt.	NA
Reddy et al., 2014 [27] Assess the impact of DFS on iron and iodine status of schoolchildren India 9 mo longitudinal intervention study 947 schoolchildren (6–15 y)	DFS Source NA Concentration NA	Hb (+0.6 * g/dL and 0.21 * g/dL, deworming and not) Anemia prevalence (−6.3% * and +1.5%, deworming and not) Ferritin NA	All the groups were also provided nutrition and health education regarding the need and importance of iodine and iron nutrition in children.	Albendazole (400 mg) given twice	NA	NA	NA
Reddy et al., 2016 [28] Assess the impact of DFS on iron and iodine status of pregnant women India 9 mo RCT 150 pregnant women (<12 weeks gestation)	DFS Ferrous sulfate 1000 ppm at 10 g/day	Hb (+0.42 * g/dL) Anemia prevalence (−10.5%) Ferritin NA	NA	NA	NA	Nutrient intake was recorded using 24 h dietary recall information.	All pregnant women were on iron, folic acid, and calcium supplements during 2nd and 3rd trimester
Vinodkumar et al., 2007 [29] Test the stability of DFS during storage and to assess its efficacy in improving the iron and iodine status in communities India 1 y multicenter, single-blind, RCT 829 family members	DFS FS monohydrate chelated with malic acid and sodium hexameta-phosphate 1 mg Fe/g salt	Hb (+1.98 * g/dL) Anemia prevalence NA Ferritin NA	NA	Dewormed at baseline, 6 mo, and 12 mo	No complaints regarding taste. People noted that the amount of salt to be added to food was less, and that food turned slightly sour when kept for more than 6 h. After use for 1 y, 98%–100% of households rated the taste and color of DFS as acceptable.	NA	NA
Wegmueller et al., 2006 [30] Test efficacy of DFS Cote d’Ivoire 6 mo, double-blind, efficacy RCT 123 iron-deficient children (5–15 y)	DFS Micronized ground FePP 3 mg Fe/g salt	Hb (0 g/dL) Anemia prevalence (+5%) Ferritin (+15 * μg/L)	In a village meeting at the beginning of the study and at each of the monthly salt distribution, it was emphasized that the salt should be used for all cooking, food preparation, and at the table.	At the baseline screening and again at 4 mo, all children received an oral dose of 400 mg albendazole	Triangle test by 18–21 untrained local adults were not able to detect differences in color, odor, or taste in either traditional staples (rice, cassava, yam, plantain) or sauces (tomato, eggplant, okra, palm nut)	Remaining salt at the end of the month was weighed and the amount of salt consumed per day per person during the period since the last distribution calculated. Calculated per capita salt consumption in the households using DFS was 4.0 +/−2.5 and 6.1 +/−4.0 g/d at 1 and 6 mo.	Median vitamin A intake was at the EAR for children 2–5 y and above the EAR for children 6–15 y, women, and men, due to high consumption of red palm oil and refined palm oil fortified with retinyl palmitate. 7% of children had vitamin A deficiency at baseline
Wenger et al., 2017 [31] Assess the extent to which increases in dietary iron consumption are related to improvements in behavioral measures of perceptual, attentional, and mnemonic function India 10 mo RCT 126 NP women (18–55 y)	DFS Micro-encapsulated FF 1.1 mg Fe/g salt	Hb (+0.1 * g/dL) Anemia prevalence (−5% *) Ferritin (+12.5 * μg/L)	NA	200 mg albendazole was administered to all eligible participants 4 wk before and 4 mo after the initial baseline blood collection	It was considered acceptable by participants in a previous study	NA	Folate deficiency (−22% *) Vitamin B12 (−14.8 * μg/L and +12% * deficiency)
Zimmermann et al., 2003 [32] Test DFS efficacy Morocco 9 mo double-blind, randomized trial 377 iodine-deficient children (6–15 y)	DFS FS hydrate encapsulated with partially hydrogenated vegetable oil 1 mg Fe/g salt	Hb (+1.4 * g/dL) Anemia prevalence (−25% *) Ferritin (+20 * μg/L)	At each monthly salt distribution, all salt used for cooking and food preparation was emphasized.	NA	Baseline acceptability interviews indicated nearly unanimous acceptance. At 8 mo, 14% of households rated the color unacceptable in the damp season. 17% reported that the salt changed the color of foods. In a triangle test, there was no significant, detectable difference in color, odor, or taste	NA	NA
Zimmermann et al., 2004 [33] Test efficacy of DFS for reducing the prevalence of iodine and iron deficiencies in children Morocco 10 mo double-blind, RCT 158 children (6–15 y)	DFS Micronized FePP 2 mg Fe/g salt	Hb (+1.5 * g/dL) Anemia prevalence (−25% *) Ferritin (+17.6 * μg/L)	NA	NA	Triangle test showed no significant difference in color, odor, or taste between the salts in any of the traditional foods	NA	NA
Kumar et al., 2007 [34] Test the efficacy of a multiple micronutrient fortified salt in improving the micronutrient status and health of schoolchildren and its effect on cognition India 1 y RCT 129 children (7–11 y)	Multiple micronutrient fortified salt (microencapsulated vitamin A, B1, B2, B6, B12, folic acid, niacin, calcium pantothenate, and iodine) Chelated FS 1 mg Fe/g salt	Hb (+0.65 * g/dL) Anemia prevalence NA Ferritin NA	NA	400 mg albendazole at baseline, after 6 mo and 1 y of intervention (end of study)	The fortified salt did not change the color or taste of any food preparation	The continuous use of the fortified salt in all the meals prepared every day was monitored. All the food prepared is consumed.	Salt contained 5 μg folic acid/g salt, 400 μg vitamin B12/kg salt, and 300 IU vitamin A/g salt. Serum vitamin A significantly increased by 4.9 μg/dL
Kumar et al., 2014 [35] Establish the efficacy of multi-micronutrient fortified salt in addressing multiple micronutrient deficiencies among children compared to nutrition education and no intervention India 8 mo RCT 528 children (5–15 y)	Multiple micronutrient fortified salt (iron, iodine, vitamin A, vitamin B12, and folic acid) Chelated FS 1 mg Fe/g salt	Hb (+0.5 g/dL) Anemia prevalence (−13.4% *) Ferritin (+10.8 * μg/L)	Prior to the start of the study, a list of locally available foods that are rich in micronutrients and recipes for was communicated. Then, every month a specific topic was chosen, and in-depth education was given on that topic.	Children in all three arms of the study were given a tablet of albendazole (400 mg) at baseline and post-intervention after 8 mo	NA	Health workers visited the homes once a month to collect leftover salt. All homes only used the salt provided by the study in the intervention group, and salt was used to cook the children all three meals and an evening snack, daily.	Salt contained 1 μg folic acid/g salt, 0.1 μg vitamin B12/g salt, and 300 IU vitamin A/g salt. Vitamin A deficiency prevalence reduced by 23.5%
Vinodkumar et al., 2009 [36] Develop a salt fortified with multiple micronutrients, test its stability, and assess its efficacy in improving the micronutrient status of schoolchildren India 1 y pre- and post-test design with experimental and control groups 245 children (5–15 y)	Multiple micronutrient fortified salt (microencapsulated vitamins A, B1, B2, B6, B12, folic acid, niacin, calcium pantothenate, and iodine) Chelated FS 1 mg Fe/g salt	Hb (+0.55 * g/dL) Anemia prevalence (−4.3%, significance NA) Ferritin NA	NA	Albendazole 400 mg at baseline, after 6 mo and after 1 y of intervention	NA	Continuous use in daily cooking was monitored. NA adherence	Salt contained 5 μg folic acid/g salt, 0.4 μg vitamin B12/g salt, and 300 IU vitamin A/g salt. There was a 5.6 μg/dL increase in serum vitamin A.
Vinodkumar et al., 2009 [37] Test efficacy of multiple micronutrient fortified salt on children India 9 mo RCT 402 children (5–18 y)	Multiple micronutrient fortified salt (vitamins A, B1, B2, B6, B12, folic acid, niacin, iron, iodine, and zinc) Chelated FS 1 mg Fe/g salt	Hb (+0.67 * g/dL) Anemia prevalence (−40% *) Ferritin (-0.12 μg/L)	NA	Albendazole (400 mg) at baseline, 4 mo, and post-trial at 9 mo	The cooking staff confirmed that the fortified salt did not change the color or taste of any food	Weighed salt leftover from the previous month to verify compliance. No food was left on the plates.	Salt contained 10 μg folic acid/g salt, 0.4 mcg vitamin B12/g salt, and 300 IU vitamin A/g salt. There was a significant 4.7 μg/dL increase in serum vitamin A and 10,129 pg/mL increase in serum B12. Serum folic acid significantly decreased by −6.28 ng/mL.
Zimmermann et al., 2004 [38] Develop a stable, efficacious salt fortified with iodine, iron, and vitamin A Morocco 10 mo double-blind, RCT 157 children (6–14 y)	Triple fortified salt (iron, vitamin A, iodine) Micronized FePP at 2 mg Fe/g salt	Hb (+1.5 * g/dL) Anemia prevalence (−26% *) Ferritin (+16.0 * μg/L)	NA	NA	Triangle test showed no significant difference in color, odor, or taste (or all three) between the fortified salts in any of the traditional foods. However, 32% noted a color change in one or more foods when the salt was added. This did not affect the overall acceptability	NA	Salt contained 60 μg vitamin A/g salt. Vitamin A deficiency decreased by 8%.
Winichagoon et al., 2006 [39] Assess the efficacy of a micronutrient-fortified seasoning powder served with a school lunch on reducing anemia and improving the micronutrient status of children Thailand 31 wk RCT 569 children (5.5–13.4 y)	Seasoning powder (zinc, iron, vitamin A, and iodine) H-reduced elemental iron encapsulated with partially hydrogenated vegetable oil 5 mg Fe/pouch	Hb (+0.31 g/dL) Anemia prevalence NA Ferritin (−11.5 μg/L)	NA	NA	NA	Teachers recorded whether the child ate “all,” “more than half,” “half,” “less than half,” or “none” of the school lunch. Adherence NA	The seasoning packet contained 270 μg of vitamin A. Fortification had no effect on serum retinol.
Longfils et al., 2008 [40] To assess the efficacy and safety of fortified fish sauce, added to daily school meals either as NaFeEDTA or as FeSO4+ citrate Cambodia 21 wk double-blinded, placebo controlled RCT 140 iron-deficiency anemic children (6–21 y)	Fish sauce NaFeEDTA or FeSO4+ citrate 1 mg Fe/mL	Hb (+0.29 * g/dL and +0.31 * g/dL, FeSO4+ citrate and NaFeEDTA) Anemia prevalence NA Ferritin (+13.5 * and +17.3 * μg/L)	NA	500 mg Mebendazole at the beginning, in the middle, and at the end of the study in addition to doses provided by the National Health Program	The taste of the school meals, corresponding to an average Cambodian fare, remained unaffected after the addition of either variety	Field workers fully supervised the ingestion of the meals. Food that was not consumed was re-weighed and recorded. All meals were fully consumed with no left-over.	NA
Van Thuy et al., 2005 [41] Evaluate the effectiveness of fortified fish sauce with for improving iron status in WRA Vietnam 18 mo double-blind, intervention with randomization by village 576 WRA	Fish sauce NaFeEDTA 9 mmol Fe/L	Hb (+0.54 * g/dL) Anemia prevalence (−16.2% *) Ferritin (+36.3 * μg/L)	NA	NA	The fortified fish sauce was well accepted by the target population over an 18 mo period	Compliance monitoring NA Mean consumption for all individuals in all villages was 18 mL/(person x d)	<8.0% of the women had vitamin A deficiency.
Chen et al., 2005 [42] Study the effectiveness of NaFeEDTA-fortified soy sauce for controlling iron deficiency in a high-risk population China 18 mo RCT 14,000 residents (3+ y)	Soy sauce NaFeEDTA 29.6 mg Fe/100 mL	Hb (M3 − 6 y +0.69 *, M7 − 18 y + 1.0 *, M19 − 54 y + 0.98 *, M55 + y + 0.88 *, W3 − 6 y + 0.75 *, W7-18 y + 1.0 *, W19 − 30 y + 1.2 *, W31-54 y + 1.56 *, W55 + y + 0.72 * g/dL) Anemia prevalence (M3-6 y − 31.9% *, M7-18 y − 36.3% *, M19-54 y -25.6% *, M55 + y − 37.8% *, W3-6 y − 26.1% *, W7 – 18 y − 40.6% *, W19 – 30 y − 44.9% *, W31 – 54 y − 42.8% *, W55 + y − 34.9% *) Ferritin (M7 − 18 y + 1.87 *, M19 − 54 y + 6.79 *, M55 + y + 2.91 *, F7 − 18 y + 1.74 *, F19 − 30 y + 1.34 *, F31 − 54 y + 1.86 *, F55 + y +3.43 * μg/L)	NA	NA	A survey of the organoleptic qualities and acceptance of the fortified/unfortified soy sauce was conducted in 187 households. Both products were considered to be of high quality and no complaints of adverse effects. The two were reported to taste the same	Food frequency questionnaires at baseline, 6, 12, and 18 mo. During the trial period, the mean soy sauce consumption increased from 14.3 to 16.4 mL/person/day in the fortified group and from 14.1 to 15.8 mL/person/day in the control group.	There were no significant differences in plasma retinol levels between the fortified and control groups. There may be a high prevalence of subclinical vitamin A deficiency in these villages. Folate and B12 deficiencies should be considered in future studies.
Huo et al., 2002 [43] Study the therapeutic effects of NaFeEDTA-fortified soy sauce on anemic students at two concentration levels China 3 mo RCT 304 iron-deficiency anemic children (11–17 y)	Soy sauce NaFeEDTA Low = 1 mg Fe/mL High = 4 mg Fe/mL	Hb (+2.03 * and +2.39 * g/dL, low and high Fe levels) Anemia prevalence NA Ferritin (+17.19 * and +15.65 * μg/L, low and high Fe levels)	NA	NA	NA	The soup was consumed under complete supervision from the teachers. At the same time, detailed information on the soy sauce consumption of each subject was recorded on a consumption sheet. Adherence NA.	NA

Notes: DFS—double-fortified salt; FePP—ferric pyrophosphate; FF—ferrous fumarate; EFF—encapsulated FF; FS—ferrous sulfate; Hb—hemoglobin; ID—iron deficiency; mo—month; NA—data or information not available or unknown; NP—non-pregnant; NL—non-lactating; RCT—randomized control trial; wk—week; WRA—women of reproductive age; y—year. * Indicates statistically significant outcome.

**Table 2:** Studies with statistically significant changes in markers of anemia and iron status.

Outcome	Children (16 Studies) ^1^			Women (Eight Studies) ^2^			Whole Population (One Study)		
	YES	NO	NA	YES	NO	NA	YES	NO	NA
Increased hemoglobin	13	3	0	6	1	1	1	0	0
Decreased anemia	10	2	4	4	3	1	0	0	1
Increased Ferritin	9	2 *	5	4	0	4	0	0	1

NA: not assessed. ^1^ Children studies include those exclusively assessing children, Chen [42] (disaggregates data based on gender and age), and Asibey-Berko [22] (assesses both women and children). ^2^ Women studies include those exclusively assessing women, Chen [42] (disaggregates data based on gender and age), and Asibey-Berko [22] (assesses both women and children). * Overall interaction was significant (due to negative changes in the control group) [37].

**Table 3:** Low-performing studies of the efficacy of iron fortification of condiments. Impact on hemoglobin, anemia, and iron status.

Category from Table 2	Study/Objective/Country/Duration and Experimental Design/Subjects	Condiment/ Fe Source/Concentration	Iron Intervention Outcomes (Hemoglobin, Anemia Prevalence, and Ferritin)	Suggested Possible Causes (Low-Performing Factors)
Did not increase hemoglobin significantly (children)	Kumar et al., 2014 [35] Establish the efficacy of multi-micronutrient fortified salt in addressing multiple micronutrient deficiencies among children compared to nutrition education and no intervention. India 8 mo RCT 528 children (5–15 y)	Multiple micronutrient fortified salt (iron, iodine, vitamin A, vitamin B12, and folic acid) Chelated FS 1 mg Fe/g salt	Hb (+0.5 g/dL, NS) Anemia prevalence (−13.4% *) Ferritin (+10.8 * μg/L)	Insignificant hemoglobin increase was not addressed by authors
Did not increase hemoglobin significantly (children) Did not decrease anemia significantly (children)	Wegmueller et al., 2006 [30] Test efficacy of DFS Cote d’Ivoire 6 mo, double-blind efficacy trial 123 iron-deficient children (5–15 y)	DFS Micronized ground FePP 3 mg Fe/g salt	No change in Hb (0 g/dL) Anemia prevalence (+5%, NS) Ferritin increased (+15 * μg/L)	High prevalence of malaria (55%) and multiple micronutrient deficiencies (B2 deficiency 66%). Up to 52% of households reported darkening of food
Did not increase hemoglobin significantly (children) Did not increase ferritin significantly (children)	Winichagoon et al., 2006 [39] Assess the efficacy of a micronutrient-fortified seasoning powder served with a school lunch on reducing anemia and improving the micronutrient status of children Thailand 31 wk RCT 569 children (5.5–13.4 y)	Seasoning powder (zinc, iron, vitamin A, and iodine) H-reduced elemental iron encapsulated with partially hydrogenated vegetable oil 5 mg Fe/pouch	No significant change in Hb (+0.31 g/dL, NS) Anemia prevalence NA No significant change in Ferritin (−11.5 μg/L, NS)	Insufficient content (5 mg/serving) and form of iron used in the intervention (Reduced elemental Fe)
Did not increase hemoglobin significantly (women)	Nair et al., 2014 [25] Test efficacy of DFS with nutrition education on pregnant, anemic mothers India 2 mo RCT 135 pregnant, anemic women	DFS Fe source NA 1 mg Fe/g salt	Hb change before and after intervention: −0.15 g/dL (NS) Anemia prevalence NA Ferritin NA	Not enough Fe intake to meet pregnancy needs during only 2 mo of intervention
Did not decrease anemia significantly (children)	Reddy et al., 2014 [27] To assess the impact of DFS on iron and iodine status of schoolchildren. India 9 mo longitudinal intervention study 947 schoolchildren (6–15 y)	DFS Source NA Concentration NA	Hb (+0.6 * g/dL and 0.21 * g/dL, deworming and not) Anemia prevalence (−6.3% * and +1.5%, deworming and not) Ferritin NA	Absence of deworming
Did not decrease anemia significantly (women)	Asibey-Berko et al., 2007 [22] Test efficacy of DFS on anemia and iodine deficiency in women and children Ghana 8 mo RCT 300 NP, NL women (15–45 y) and 157 children (1–5 y)	DFS/ FF/ 1 mg Fe/g salt	No Hb post-treatment data Anemia prevalence: Children: −21.7% * Women: +3.3% Ferritin NA	Significant increase of anemia in the control group. Uneven baseline prevalence of anemia. Darker color of DFS. Women reported the darkening of plantains when frying
Did not decrease anemia significantly (women)	Haas et al., 2014 [24] Test efficacy of DFS in reducing ID in WRA India 7.5–9 mo RCT 212 NP women (18–55 y)	DFS Micro-encapsulated FF 1.1 mg Fe/g salt	Hb (+0.24 * g/dL) Anemia prevalence (+1%) Ferritin (+0.13 * log10 μg/mL), 34% increase.	High prevalence of folate and B12 deficiencies. High prevalence of elevated MCV (25%)
Did not decrease anemia significantly (women)	Reddy et al., 2016 [28] Assess the impact of DFS on iron and iodine status of pregnant women. India 9 mo RCT 150 pregnant women (<12 weeks gestation)	DFS Ferrous sulfate 1000 ppm at 10 g/day	Hb (+0.42 * g/dL) Anemia prevalence (−10.5%) Ferritin NA	Significance of anemia not addressed by authors
Did not increase ferritin significantly (children)	Vinodkumar et al., 2009, Int. J Vit Nut Res [37] Test efficacy of multiple micronutrient fortified salt on children India 9 mo RCT 402 children (5–18 y)	Multiple micronutrient fortified salt (vitamins A, B1, B2, B6, B12, folic acid, niacin, iron, iodine, and zinc) Chelated FS 1 mg Fe/g salt	Hb increased (+0.67 * g/dL) Anemia prevalence decreased (−40% *) No change in Ferritin (−0.12 μg/L) NS	Uneven prevalence of anemia and ferritin levels at baseline. Possible adverse interaction with zinc absorption

Notes: DFS—double-fortified salt; FePP—ferric pyrophosphate; FF—ferrous fumarate; EFF—encapsulated FF; FS—ferrous sulfate; Hb—hemoglobin; ID—iron deficiency; mo—month; NA—data or information not available or unknown; NP—non-pregnant; NL—non-lactating; RCT—randomized control trial; wk—week; WRA—women of reproductive age; y—year. * Indicates statistically significant outcome.

**Table 4:** High-performing studies of the efficacy of iron fortification of condiments. Studies were selected because all iron indicators (hemoglobin, anemia prevalence, and ferritin) demonstrated a positive impact.

Study/Objective/Country/ Duration and Experimental Design/Subjects	Condiment/Fe source/Concentration	Iron Intervention Outcomes (Hemoglobin, Anemia Prevalence, and Ferritin)	Reported Possible Causes of Success	Quality Assessment Score and Effect Size
Andersson 2008 [21] Compare the efficacy of DFS with FePP and EFF India 10 mo RCT 458 rural children (5–15 years)	DFS Micronized ground FePP or encapsulated FF 2 mg Fe/g salt	Hb (+0.6 * and +0.8 * g/dL, FePP and FF) Anemia prevalence (−9.1% * and −10.1% *, FePP and FF) Ferritin (+6.4 * and +4.9 * μg/L, FePP and FF)	Reiteration of health effects and instructions at each salt distribution Parasitic treatment Assessed sensory Compliance monitored Vitamin A supplements	Strong quality assessment 0.24 and 0.23 effect sizes, encapsulated FF and micronized FePP, respectively
Wenger 2017 [31] To assess the extent to which increases in dietary iron consumption are related to improvements in behavioral measures of perceptual, attentional, and mnemonic function. India 10 mo RCT 126 NP women (18–55 y)	DFS Micro-encapsulated FF 1.1 mg Fe/g salt	Hb (+0.1 * g/dL) Anemia prevalence (−5% *) Ferritin (+12.5 * μg/L)	Parasitic treatment Assessed sensory Assessed folate and vitamin B12 deficiencies	Strong quality assessment 0.13 effect size
Zimmermann 2003 [32] To test DFS efficacy. Morocco 9 mo double-blind, RCT 377 iodine-deficient children (6–15 y)	DFS FS hydrate encapsulated with partially hydrogenated vegetable oil 1 mg Fe/g salt	Hb (+1.4 * g/dL) Anemia prevalence (−25% *) Ferritin (+20 * μg/L)	Assessed acceptability and sensory	Strong quality assessment 0.91 effect size
Zimmermann 2004 [33] To test the efficacy of DFS for reducing the prevalence of iodine and iron deficiencies in children. Morocco 10 mo double-blind, RCT 158 children (6–15 y)	DFS Micronized FePP 2 mg Fe/g salt	Hb (+1.5 * g/dL) Anemia prevalence (−25% *) Ferritin (+17.6 * μg/L)	Assessed acceptability and sensory	Strong quality assessment 1.36 effect size
Van Thuy 2005 [41] To evaluate the effectiveness of fortified fish sauce with for improving iron status in WRA. Vietnam 18 mo double-blind, intervention with randomization by village 576 WRA	Fish sauce NaFeEDTA 9mmol Fe/L	Hb (+0.54 * g/dL) Anemia prevalence (−16.2% *) Ferritin (+36.3 * μg/L)	Assessed acceptability Assessed adherence Assessed vitamin A deficiency prevalence	Strong quality assessment 0.46 effect size
Chen 2005 [42] To study the effectiveness of NaFeEDTA-fortified soy sauce for controlling iron deficiency in a high-risk population. China 18 mo RCT 14,000 residents (3 + y)	Soy sauce NaFeEDTA 29.6 mg Fe/100mL	Hb (M3 − 6 y + 0.69 *, M7 − 18 y + 1.0 *, M19 − 54 y + 0.98 *, M55 + y + 0.88 *, W3-6 y + 0.75 *, W7 − 18 y + 1.0 *, W19 − 30 y + 1.2 *, W31 − 54 y + 1.56 *, W55 + y + 0.72 * g/dL) Anemia prevalence (M3 − 6 y − 31.9% *, M7 − 18 y − 36.3% *, M19 − 54 y − 25.6% *, M55 + y − 37.8% *, W3 − 6 y − 26.1% *, W7 − 18 y − 40.6% *, W19 − 30 y − 44.9% *, W31 − 54 y − 42.8% *, W55 + y − 34.9% *) Ferritin (M7 − 18 y +1.87 *, M19 − 54 y + 6.79 *, M55 + y + 2.91 *, F7 − 18 y + 1.74 *, F19 − 30 y + 1.34 *, F31 − 54 y + 1.86 *, F55 + y + 3.43 * μg/L)	Assessed sensory and acceptability Monitored compliance and adherence Assessed plasma retinol levels	Strong quality assessment F19 − 30 y 0.06, F31 − 54 y 0.50, F3 − 6 y 0.40, F55 + y 0.34, F7 − 18 y 0.34, M19 − 54 y 0.51, M3 − 6 y 0.51, M55 + y 0.41, M7 − 18 y 0.26
Kumar 2014 [35] To establish the efficacy of multi-micronutrient fortified salt in addressing multiple micronutrient deficiencies among children compared to nutrition education and no intervention. India 8 mo RCT 528 children (5–15 y)	Multiple micronutrient fortified salt (iron, iodine, vitamin A, vitamin B12, and folic acid) Chelated FS 1 mg Fe/g salt	Hb (+0.5 g/dL) Anemia prevalence (−13.4% *) Ferritin (+10.8 * μg/L)	Education component Parasitic treatment Monitored compliance and adherence Vitamin A deficiency prevalence reduced	Strong quality assessment 0.40 effect size
Zimmermann 2004 [38] To develop a stable, efficacious salt fortified with iodine, iron, and vitamin A. Morocco 10 mo double-blind, RCT 157 children (6-14 y)	Triple fortified salt (iron, vitamin A, iodine) Micronized FePP at 2 mg Fe/g salt	Hb (+1.5 * g/dL) Anemia prevalence (−26% *) Ferritin (+16.0 * μg/L)	Assessed sensory and acceptability Vitamin A deficiency prevalence reduced	Strong quality assessment 1.55 effect size

Notes: DFS—double-fortified salt; FePP—ferric pyrophosphate; FF—ferrous fumarate; EFF—encapsulated FF; FS—ferrous sulfate; Hb—hemoglobin; ID—iron deficiency; mo—month; NA—data or information not available or unknown; NP—non-pregnant; NL—non-lactating; RCT—randomized control trial; wk—week; WRA—women of reproductive age; y–year. * Indicates statistically significant outcome.

## Supplementary Materials

The following are available online at https://www.mdpi.com/2072-6643/12/2/275/s1. Table S1: Quality assessment of articles included in this review.
