# Supplementary material for: Performance Factors Influencing Efficacy and Effectiveness of Iron Fortification Programs of Condiments for Improving Anemia Prevalence and Iron Status in Populations: A Systematic Review
Source: Nutrients. 2020 Jan 21;12(2):275. doi: 10.3390/nu12020275 (PMC7070935; doi:10.3390/nu12020275)
Supplement: Supplementary file 1 [file nutrients-12-00275-s001.pdf]

Table S1. Quality assessment of articles included in this Review. The final quality rating was based on the number of "No" answers to the 10 quality criteria as well as the number of unclear (Ucl) answers to the sub-questions within these criteria (data not shown).

Rating:
- Strong ● (green)
- Moderate ● (grey)
- Weak ● (red)

	Andersson 2008	Bathla 2016	Haas 2014	Rajagopalan 2000	Reddy 2014	Reddy 2016	Vinodkumar 2007	Wenger 2017	Zimmermann 2003	Zimmermann 2004	Longfils 2008	Van Thuy 2005	Kumar 2007	Kumar 2014	Vinodkumar 2009_1	Vinodkumar 2009_2	Chen 2005	Huo 2002	Zimmermann 2004	Winichagoon 2006	Wegmuller 2006	Nair 2014	Asibey-Berko 2007
**Overall Quality Rating**	Strong	Weak	Strong	Moderate	Weak	Moderate	Moderate	Strong	Strong	Strong	Strong	Strong	Weak	Strong	Moderate	Moderate	Strong	Moderate	Strong	Strong	Strong	Weak	Moderate
1. Would implementing the studied intervention or procedure (if found successful) result in improved outcomes for the patients/ clients/ population group?	Yes	Yes	Yes	Yes	Yes	Yes	Yes	Yes	Yes	Yes	Yes	Yes	Yes	Yes	Yes	Yes	Yes	Yes	Yes	Yes	Yes	Yes	Yes
2. Did the authors study an outcome or topic that the patients/ clients/ population group would care about?	Yes	Yes	Yes	Yes	Yes	Yes	Yes	Yes	Yes	Yes	Yes	Yes	Yes	Yes	Yes	Yes	Yes	Yes	Yes	Yes	Yes	Yes	Yes
3. Is the focus of the intervention or procedure or topic of study a common issue of concern to dietetics practice?	Yes	Yes	Yes	Yes	Yes	Yes	Yes	Yes	Yes	Yes	Yes	Yes	Yes	Yes	Yes	Yes	Yes	Yes	Yes	Yes	Yes	Yes	Yes
4. Is the intervention or procedure feasible?	Yes	Yes	Yes	Yes	Yes	Yes	Yes	Yes	Yes	Yes	Yes	Yes	Yes	Yes	Yes	Yes	Yes	Yes	Yes	Yes	Yes	Yes	Yes
1. Was the research question clearly stated?	Yes	Yes	Yes	Yes	Yes	Yes	Yes	Yes	Yes	Yes	Yes	Yes	Yes	Yes	Yes	Yes	Yes	Yes	Yes	Yes	Yes	Yes	Yes
2. Was the selection of study subjects/patients free from bias?	Yes	Yes	Yes	Yes	Ucl	Ucl	Yes	Yes	Yes	Yes	Yes	Yes	Ucl	Yes	Ucl	Ucl	Yes	Yes	Yes	Yes	Yes	Ucl	Yes
3. Were study groups comparable?	Yes	Yes	Yes	Yes	Ucl	Yes	Ucl	Yes	Yes	Yes	Yes	Yes	Yes	Yes	Ucl	Ucl	Yes	Yes	Yes	Yes	Yes	Ucl	Yes
4. Was method of handling withdrawals described?	Yes	Yes	Yes	Yes	Yes	Yes	Yes	Yes	Yes	Yes	Yes	Yes	Yes	Yes	Yes	Yes	Yes	Ucl	Yes	Yes	Yes	Yes	Yes
5. Was blinding used to prevent introduction of bias?	Yes	No	Yes	Yes	No	No	Ucl	Yes	Yes	Yes	Ucl	Ucl	No	No	No	No	Yes	No	Yes	No	Yes	No	Yes
6. Were intervention/therapeutic regimens/exposure factor or procedure and any comparison(s) described in detail?	Yes	Yes	Yes	Yes	Yes	Yes	Yes	Yes	Yes	Yes	Yes	Yes	Yes	Yes	Yes	Yes	Yes	Yes	Yes	Yes	Yes	No	Yes
7. Were outcomes clearly defined and the measurements valid and reliable?	Yes	Ucl	Yes	Yes	Yes	Yes	Yes	Yes	Yes	Yes	Yes	Yes	Yes	Yes	Yes	Yes	Yes	Yes	Yes	Yes	Yes	Yes	Yes
8. Was the statistical analysis appropriate for the study design and type of outcome indicators?	Yes	No	Yes	No	Yes	No	No	Yes	Yes	Yes	Yes	Yes	Yes	Yes	Yes	Yes	No	No	Yes	Yes	Yes	No	Yes
9. Are conclusions supported by results with biases and limitations taken into consideration?	Yes	Ucl	Yes	Yes	Yes	Yes	Yes	Yes	Yes	Yes	Yes	Yes	Yes	Yes	Yes	Yes	Yes	Yes	Yes	Yes	Yes	Yes	Yes
10. Is bias due to study's funding or sponsorship unlikely?	Yes	Yes	Yes	Yes	Yes	Yes	Yes	Yes	Yes	Yes	Yes	Yes	Yes	Yes	Yes	Yes	Yes	Yes	Yes	Yes	Yes	Yes	Yes
